# Studying Smaller and Neglected Organisms in Modern Evolutionary Venomics Implementing RNASeq (Transcriptomics)—A Critical Guide

**DOI:** 10.3390/toxins10070292

**Published:** 2018-07-16

**Authors:** Björn Marcus von Reumont

**Affiliations:** 1 Justus Liebig University of Giessen, Institute for Insect Biotechnology, Heinrich Buff Ring 58, 35392 Giessen, Germany; bmvr@arcor.de; 2 Natural History Museum, Department of Life Sciences, Cromwell Rd, London SW75BD, UK

**Keywords:** evolutionary venomics, transcriptomics, proteomics, pooled samples, assembly, read mapping, toxin expression level

## Abstract

Venoms are evolutionary key adaptations that species employ for defense, predation or competition. However, the processes and forces that drive the evolution of venoms and their toxin components remain in many aspects understudied. In particular, the venoms of many smaller, neglected (mostly invertebrate) organisms are not characterized in detail, especially with modern methods. For the majority of these taxa, even their biology is only vaguely known. Modern evolutionary venomics addresses the question of how venoms evolve by applying a plethora of -omics methods. These recently became so sensitive and enhanced that smaller, neglected organisms are now more easily accessible to comparatively study their venoms. More knowledge about these taxa is essential to better understand venom evolution in general. The methodological core pillars of integrative evolutionary venomics are genomics, transcriptomics and proteomics, which are complemented by functional morphology and the field of protein synthesis and activity tests. This manuscript focuses on transcriptomics (or RNASeq) as one toolbox to describe venom evolution in smaller, neglected taxa. It provides a hands-on guide that discusses a generalized RNASeq workflow, which can be adapted, accordingly, to respective projects. For neglected and small taxa, generalized recommendations are difficult to give and conclusions need to be made individually from case to case. In the context of evolutionary venomics, this overview highlights critical points, but also promises of RNASeq analyses. Methodologically, these concern the impact of read processing, possible improvements by perfoming multiple and merged assemblies, and adequate quantification of expressed transcripts. Readers are guided to reappraise their hypotheses on venom evolution in smaller organisms and how robustly these are testable with the current transcriptomics toolbox. The complementary approach that combines particular proteomics but also genomics with transcriptomics is discussed as well. As recently shown, comparative proteomics is, for example, most important in preventing false positive identifications of possible toxin transcripts. Finally, future directions in transcriptomics, such as applying 3rd generation sequencing strategies to overcome difficulties by short read assemblies, are briefly addressed.

## 1. What is Modern Evolutionary Venomics?

**Figure 1:**
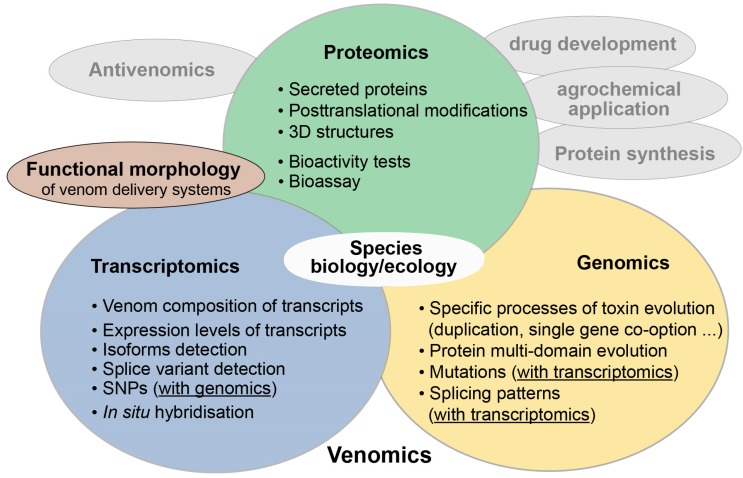
Modern evolutionary venomics. The integrative approach combines a plethora of different new -omics methods (colored circles) to study venom evolution. Their synthesis enables a detailed insight into venom biology, evolutionary processes that drive toxin evolution, but also the ecology and evolution of venomous species. Illustrated in grey are other fields in venomics such as antivenomics to develop antidotes, but also drug development and agrochemical applications. These applied areas are linked to activity tests and bioassays of (putative) toxins, which represent special areas in proteomics. Functional morphology becomes more and more important based on state-of-the-art 3D reconstructions to study different toxin expression and internal structures of venom delivery systems. In this case, morphology is strongly interwoven with transcriptomics, for example to apply in situ hybridization or fluorescence marker to identify toxin expression locations.

The term venomics was first coined in 2004 by Calvete and colleagues who applied proteomic analyses on snake venoms [1,2]. Today the term venomics mostly labels studies on venom in which a plethora of new -omics technologies is applied, such as transcriptomics, proteomics, antivenomics and genomics, often combined in an integrative approach [3,4]. New complementary methods in functional morphology and applied proteomics to test toxin activity for antivenom research, pharmaceutical or agrochemical applications complete the methodological arsenal in venomics [3,5,6,7,8,9,10,11] (see also Figure 1). The term ‘evolutionary venomics’ suggested here implies that a combined methodological approach is utilized to better comprehend the evolution of venom systems and their components. It also includes all aspects of the ecology and biology of the studied venomous organisms. This connotation is similar to the one used rather early in a Toxicon editorial from 2006 that announced a larger venomics project. The aim was to complementarily combine genomic data with proteomics and transcriptomics for a few selected venomous taxa [12] to understand their venom evolution.

The biggest chance of the combined, comparative approach in evolutionary venomics is to understand unprecedented details of toxin composition, processes of venom evolution, but also the biology of venomous species, which are in many cases not well known or unclear [3,13,14,15]. These modern methods facilitate more extensive studies of smaller, previously neglected venomous taxa [3,4,16]. Invertebrates house many species that are known from observation to be possibly venomous, but most of their toxins remain untapped, which is also reasoned in their small size. In particular, insects are rather small organisms and their venom systems were difficult to study until modern -omics technologies provided the platform to develop feasible comparative analyses of small-scale venom systems [4,16]. In this text, the term “small and neglected organisms” is mostly referred to pancrustacean taxa (most examples derive from work on robber flies or remipede crustaceans), but it can be extended to all small and rare invertebrates or other organisms.

## 2. Transcriptomics—One Major Pillar in Evolutionary Venomics

Transcriptome sequencing and analyses (often synonymously referred to as RNASeq [17]) rapidly advanced in the last decades not only in the field of human biology or medicine, but also in molecular phylogenetics, where transcriptome data is used to infer processes of species evolution by phylogenomics [18,19,20,21]. Methodological insights were gained from large scale sequencing projects such as the Human Genome Project and its derivate platform ENCODE [22], 5000 arthropod genomes (i5k) [23], Genome 10K (10,000 vertebrate genomes [24]) and GIGA (Global invertebrate genomics alliance [25]). The recent insect transcriptome consortium (1KITE) investigates insect evolution by sequencing over 1000 insect transcriptomes [26]. In parallel, the sequencing chemistry improvements over time allowed deeper sequencing with better coverage, simultaneously accompanied with decreasing amounts of needed species tissue. This general evolution of next generation sequencing (NGS) is described in several books and reviews, also comparing the different NGS sequencing platforms starting from cloned ESTs using agar plates and ending with 3rd generation platforms like Oxford Nanopore [18,27,28,29,30,31].

The venom evolution of smaller, neglected organisms is per se difficult to investigate because they are normally hard to collect, even harder to rear and additionally often very small. In most cases, these features unite. With the now established NGS Illumina platform, transcriptome work on these taxa became much easier, also because it can operate with lower quantities of RNA material to unravel, on a first level, putative venom protein transcripts. A comparative aspect is important in evolutionary venomics to see how evolutionary processes differ between lineages and which adaptations might be taxon specific, as well as draft a more general picture of venom evolution [3,4]. For this reason, a larger variety of taxa needs to be studied. In particular, neglected organisms frequently exhibit unique characteristics that raise a particular interest, be it their phylogenetic position or their expected peculiar toxin components. Remipede crustaceans, for example, are likely the sister group to insects and the first described predatory venomous crustaceans [32,33,34,35]. Their venoms could also give new perspectives and implications regarding venom evolution in pancrustaceans (crustaceans + Hexapoda [32]). Unfortunately or luckily—depending on the collectors’ perspective—remipedes occur in small numbers in marine, anchialine caves that are not easy to access. A terrestrial example are robber flies, which were suspected since the 19th century to have strong neurotoxic components because some species are specialized in hunting down well-defended prey, such as dragonflies or hymenopterans [3,5]. Robber flies equally occur rather solitarily and a successful rearing or captivity is difficult even for a short time.

Following well-established protocols is often challenging when transcriptome and proteome-based venom gland samples are obtained from small and rare taxa. It is sometimes even impossible to achieve under controlled laboratory conditions. This is in strong contrast to better studied organisms such as snakes, spiders, cone snails, assassin bugs or scorpions [6,9,36,37,38,39]. As a consequence, methodological limitations often occur that demand an assessment on how they might impact on the addressed hypotheses of venom evolution in the respective, neglected taxon. In particular, if one main (or even sometimes only [40,41]) strategy is to rely on transcriptomics, subsequent conclusions need to be drawn carefully. This article provides a very generalized “template” processing flow for RNASeq analyses in evolutionary venomics with a focus on smaller neglected organisms and related methodological issues that can be adapted for own project strategies. Advantages and disadvantages of transcriptomics are discussed and possibilities to adjust or diversify analyses are flagged. Some of these are linked to (crucial) complementary proteomic or genomic data.

## 3. Theoretical Considerations from Collection to Sequencing

### 3.1. Implications from Pooled Samples of Small, Neglected Organisms

**Figure 2:**
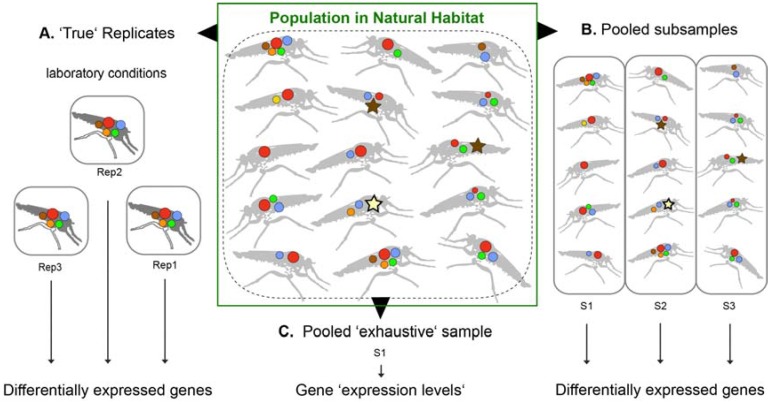
Sampling strategies to apply toxin expression analyses with transcriptomics and proteomics for small and neglected species, in this case robber flies. Differently colored circles symbolize differently expressed proteins in the venom gland system. Star-like symbols represent co-factors and house keeping genes involved in the venom synthesis that are expected to be highly expressed alongside toxins too, when venom cocktails are replenished (or glands regenerated) after a sting. The established framework for differentially expressed gene analyses demands several replicates to gain statistical power (**A**). However, rearing specimens under same conditions safeguarding their similar physiological state of the venom glands ranges from very hard to impossible for many smaller, neglected organisms. Alternative strategies are to pool subsamples (similar to biological samples) if enough specimens can be collected (**B**), or to pool tissue from all collected individuals in one exhaustive sample (**C**). If laboratory conditions cannot be achieved, one exhaustive sample better covers the whole range of venom composition from unknown physiological venom gland states. In this case, a conservative interpretation and comparative proteomics analyses to identify proteins that are actually injected into another organism are crucial. As shown in (**B**), sub-samples with only a few individuals can result by chance in extremely different pictures of toxin and protein compositions reasoned by diverse physiological states of venom glands in natural conditions, which makes differential gene expression analyses partly difficult.

Neglected venomous organisms often resemble a limited source, in most cases only few and very small specimens can be collected. Thus, venom gland tissue for transcriptomics and crude venom liquid for proteomics are on a regular base pooled from several individuals to gain enough material for subsequent analyses. Often, pooling results in only one total sample. A clear disadvantage of one pooled sample with no replicates is that established analyses of differentially expressed genes within a robust statistical framework with at least three replicates is not possible [42,43,44]. It is argued here that if the general venom composition of a neglected taxon is the focus of the study, and not the individual differences between populations or specimens, the approach of using one sample only (including several specimens) is feasible—if only general conclusions are made (see also Figure 2).

In this context, it has to be considered that venom glands exhibit different physiological states after and before a sting or bite, in which the expressed venom proteins vary [45]. Thus, the best practice is to keep all specimens in the laboratory for a few days to “pre-milk” or stimulate venom ejection directly after capturing once or several times simultaneously for all specimens to ensure similar physiological states of the venom glands. After several days, the venom gland proteins are equally replenished (or regenerated) in all individuals and the venom proteins are likely most highly expressed. The crude venom should be preserved now for comparative proteomic work. Then, the tissue of the glands can be extracted and preserved for transcriptomics. A critical aspect is that few studies suggest that the time a depleted venom gland needs to replenish is variable between species, could be dependent on temperature or age of specimens, and might differ among toxin classes [45,46]. These results imply that the best time to extract/milk venom should be tested for each neglected organism if that is achievable.

However, for difficult to collect species, it is often impossible to breed or to keep specimens under controlled conditions even for a few days. In that case, one larger sample, which includes more individuals, counter-balances (unknown) different physiological states of venom glands in wild specimens (see Figure 2). The practice to pool many individuals in “exhaustive” samples potentially blurs and “normalizes” intraspecific, spatial or gender specific differences in toxin compositions, which can be of interest or even advantage if general conclusions on the venom of a new species are the aim of the study. However, a side effect is that if specimens are pooled, highly expressed genes might be specific for the condition of each individual. If too few specimens are pooled, for example, if biological replicates are planned, this effect might have a larger impact and false positive differently expressed genes are then discussed. A larger and broader sampling of individuals results in a statistically more representative venom for a species. It is argued and recommended here that, if a “normalization” of venom glands is not to be accomplished, exhaustive samples should rather be taken from wild species if possible. Some of these aspects were already discussed in the context of snake venom variability and antivenom strategies [47]. To produce effective antidotes, local variations as well as the species typical toxin components are crucial to consider and to know. Generally, venom variation is probably best characterized for snakes—see, for example, [47,48,49,50,51]. Only a few recent studies on invertebrates discuss the fact that intraspecific venom variations can be surprisingly extensive [52,53,54,55,56]. A separation of genders is always advantageous and it is suggested to eliminate or reduce gender specific bias, which is not yet well studied in neglected venomous organisms. Most insights on gender specific venom variation are gained by analyses of snake and spider venoms [57,58,59,60,61,62,63,64].

When working with insects or small invertebrates, a similar milking procedure known from snakes is not applicable. A method of choice in most cases for invertebrates is the electro stimulation of venom glands or muscles that forces the ejection of the crude venom. Garb and colleagues describe this maneuver in detail for spiders, using a foot pedal regulated electro stimulator model [36]. Walker et al. recently presented a similar technique for assassin bugs [65]. An alternative low cost electro stimulator version that is particularly designed for small arthropods and invertebrates is based on an Arduino microcontroller board [66]. On field trips when mobility is needed, a power plug or battery operated power supply with constant voltage settings attached to specially isolated forceps might be favored over a stationary electro stimulator source [67]. If species are milked electrically, it needs to be considered in the experimental setup and project goals that ejected venom can vary in its composition (or even includes non-venom contaminants) compared to other milking (or venom extraction) methods, which can be an advantage or disadvantage, depending on the goal of the experiment [68,69,70,71].

### 3.2. Advantages of Dissecting the Whole Venom Delivery System and Its Downside

For several small organisms, the only practical way is to dissect the venom delivery system completely as soon and as fast as possible. Robber flies are hard to electro-stimulate because many species are rather small, but, more importantly, their venom glands are difficult to access, being located centrally in the thorax and linked to stomach pumps and muscles [5]. An internal contamination of the venom by gut content is risked by electro-stimulation of their muscle systems. Remipede crustaceans come with additional hurdles as marine organisms. In both cases, the best strategy is to immediately anesthetize and kill the specimens, and then to dissect the gland system, which can be squeezed out to preserve the crude venom for proteomics and the tissue for transcriptomics. Wherever applicable, gland tissue and crude venom liquid of each individual should be complementarily analyzed. Depending on the species and situation (in the field for example), it might be difficult to process all samples in a short time, in sterile conditions and on ice to prevent any degradation of proteins or RNA. All protocols and supply chains for materials needed in the field should be tested and established beforehand.

The dissection of the whole venom delivery system (gland and duct) increases the chance to recover a rather complete picture of the full toxin arsenal used by a species. Recent studies show that employed venom cocktails for predatory or defensive purposes can vary and even the expression location within the gland might be different for specific toxins [6,37,72]. In the case of being milked, specimens might rather secrete and express a fraction of the venom cocktail instead of fully emptying their glands [71]. The downside of a dissection of the complete venom gland system is that no conclusions about “predatory” and “defensive” venom variation can be drawn. This aspect is only addressed in a very careful laboratory setup, in which reactions of specimens can clearly be stimulated in a distinctive manner [6,37,65]. These considerations about milking techniques mainly concern proteomic analyses based on secreted venom proteins, but of course also need to be considered for the transcriptomic approach and for complementary analyses. The conditions when and how transcriptomic and proteomic samples were taken are fundamental for interpretation. A capital aspect is that transcripts from whole venom delivery systems always include non-venom related genes such as house keeping genes, translation factors, etc. This generally bears the risk for a false over-interpretation of putative toxin diversity (or venom proteins in general) based on transcriptomics [53]. Complementary protein data, which should be the baseline to identify secreted proteins and peptides, decreases this effect, and, in some cases, complementary body tissue samples can help to distinguish gland unique transcripts.

### 3.3. Practical Thoughts for the Dissection of Glands, Sample Preservation and Sequencing

The gland systems of small invertebrates can be dissected in small glass dishes with sterile TBE buffer. For proteomics, the crude venom should be preserved in proteinase inhibitor buffer if no cooling chain is guaranteed to prevent degradation, while the tissue for transcriptomics is stored in RNAlater. A direct processing on ice or freezing of samples until they are processed is always the favored solution, yet, for some organisms, this is often not feasible [34]. RNAlater for transcriptomics and protein inhibitor cocktails are workable alternatives [5,33,34]. However, if proteins are stored in proteinase inhibitor cocktails (for example CompleteUltra tablets from ROCHE, Mannheim, Germany), it needs to be considered that subsequent activity tests are biased or even impossible. Unfortunately, the single components of commercial buffer solutions are kept a secret by the companies.

RNA extraction is often outsourced to a sequencing company, but, in any case, extraction protocols should be tested beforehand. RNA micro extractions of venom glands from robber flies and remipedes were successfully conducted with variations from standard Trizol extraction protocols [5,34]. Using smaller solution quantities and tubes, but also a cordless motor pellet or micro pestle to break up tissue thoroughly with a sterile micro pestle, improves the extraction quality. Small amounts of tissue samples can be compensated in some cases by the application of special low quantity library protocols, such as Universal Plus mRNA (NuGEN, San Carlos, CA, USA), instead of the standard Illumina TruSeq kit (Illumina TruSeq kit, San Diego, CA, USA). However, it should be considered that a different library sample preparation might have consequences for downstream analyses, for example a more difficult identification of low expressed splicing variants linked to smaller insert sizes [73]. Depending on the number of samples that are sequenced a mis-assignment of reads, for example caused by cross-contamination during laboratory work, can be prevented with double indexed libraries [74].

One future direction in transcriptome de novo sequencing might be the application of long read backbones that are generated with 3rd generation platforms, such as Oxford Nanopore [75] or Pacbio [76]. Both platforms are capable of generating sequencing reads of a few hundred thousand base pairs. Pacbio applies a single molecule real time approach (SMRT) in which a DNA molecule is attached to an immobilized polymerase molecule at the bottom of a nano-tube [76]. The setting allows such a sensitive recording of the light emission during sequencing that even methylations can be differentiated. Oxford Nanopore uses a different approach utilizing a Nanopore attached to a membrane. If single DNA molecules are pulled through this pore by auxiliary proteins, electric pulses allow the identification of each base [75]. The long reads generated by both 3rd generation platforms can subsequently be merged with shorter Illumina based reads; currently, 150 bp are the standard for Illumina HiSeq/NextSeq or 250/300 bp on the Illumina MiSeq platform. MiSeq reads with 300 bp read length demand a critical consideration, as personal experience showed that the read quality generally drops dramatically well before 250 bp. Both 3rd generation sequencing platforms still exhibit a large sequencing error rate of +/−10 percent. This demands either higher sequence coverage or complementary sequenced Illumina short reads for a correction. This hybrid sequencing approach, already being used in genomics, would eliminate several errors that occur in the assembly process, and the longer reads would improve the overall quality of transcriptome and downstream analyses (see the next paragraph). An RNA based library preparation for long read sequencing would probably demand some protocol adaptations to normalize read abundance and to prevent too many identical long reads for overexpressed transcripts. A downside is that both 3rd generation sequencing platforms are still noticeably more expensive compared to Ilumina based sequencing.

## 4. Transcriptome Analysis and Its Complexity

### 4.1. Raw Read Filtering (Read Pre-Processing)

After retrieving the raw data from the sequencer all reads need to be pre-processed before they can be assembled to contigs (consensus transcript sequences from overlapping and merged reads). This preprocessing (or trimming) clips and excludes read parts that contain technical or contaminant sequences such as adapters from the cDNA library. In addition, sequence parts towards the 5′ and 3′ ends with low quality base calling, also referred to as phred values (reflecting an accepted error rate of wrong nucleotides [31]), are excluded. Generally, the 3′ end shows higher proportions of low quality. Finally, surviving reads of a minimum length are retained and selected for assembly.

In several studies, it was demonstrated that the quality of the read filtering processes affects and improves the assembly [77,78,79,80]. Thus, the reads need to be filtered with awareness of what chosen programs exactly do and how they perform on their own, respective data. Major variables are (1) read length and (2) phred quality value:

(1) Including longer reads increases the assembly performance because orthologous genes are better identified. This effect saturates when read lengths reach a certain threshold. However, this threshold seems sample and taxon dependent, so a general recommendation is not possible (~150 bp for tested human and mouse, and ~75 bp for yeast) [81]. One suggestion is to filter the data multiple times with different read lengths to assess its impact on finally excluded data. Depending on the used sequencing platform and general sequencing length, the impact could be severe. The goal should be to include reads with the longest possible length.
(2) Similarly, the choice of phred quality values is a trade-off between not excluding too many raw reads but retaining as many as possible good quality reads [77]. The phred-value impacts stronger on resulting quality of RNASeq data than on DNA based genome sequencing and is shown to possibly affect later gene expression results [82]. Illumina data should be filtered with a phred value of 30 or more, a phred value of 30 allows for an error rate of 99.9% (one erroneous base per 1000 bases can still be a lot depending on the sequence depth).

To make it more complex, the best settings for the read trimming and preprocessing can vary between taxa. To finally decide on the trimming, the performance of several test runs is recommended. A comparison between different trimming tools might be considered; several benchmarking studies (only shown after 2014) give an indication of appropriate choices for trimming tools [78,80,83,84]. The results can then be inspected and compared for example with FastQC or its latest implementation in the FastQC dashboard [85] before assembly is started.

### 4.2. De Novo Transcriptome Read Assembly

Today, a variety of assembly software is available for RNA based NGS data and several comparative performance reviews have been published [86,87,88], partly in larger collaborative efforts, such as the assemblathon platform [19,20]. The widely used assembler Trinity [87,89] performs well overall compared to different assemblers and offers several tools for downstream analyses, but in some cases is outperformed by other software programs [86,90]. A recent developed wrapper pipeline DRAP combines Trinity and Oases to improve assembly performance [91]. In the recent study by Holding and colleagues, the performance of assemblers to identify toxin transcripts from different venom gland tissue samples is comparatively tested, including Trinity, SPAdes, NGen14, Soapdenovo-Trans, Ngen14 and their in-house tool Extender [90]. One result was that Extender and NGen14 outperform the other tested software regarding the identification of toxin transcripts.

Most current assemblers rely on the k-mer approach in which short reads are broken down in even shorter sequence fragments, so called k-mers [92,93]. These k-mers of all transcripts are then connected stepwise in de Bruijn graphs, which are used by the assembler to reconstruct consensus sequences (contigs) based on the graph calculations (see the review with extensive overview graphics from Martin and Wang [94]). The k-mer approach is also the reason that de novo assembly is especially sensitive to sequencing errors that might induce wrong graph connections, dead connection ends or alternative loops, which all end in alternative transcripts [18,94].

In general, the quality of transcriptome assemblies is not easy to evaluate and results are often affected by heterogeneous transcriptome data [95]. A comparison of results from different assemblers for a de novo transcriptome is one possible strategy [34]. If commercial GUI based (graphical user interface) tools are used, a careful check of parameter settings, but also a performance test against command line based but probably memory and hardware demanding assemblers is advised [96]. Possible and common assembly errors, for example chimeric transcripts (parts of two transcripts are merged) or collapsed family gene variants (transcripts from different genes are merged) can be assessed and compared for different assemblies by Transrate [97]. However, the statistics to compare different assemblies are somewhat difficult and often not very meaningful, particular for putative toxin transcripts [90]. The practice to estimate the completeness of a transcriptome by matching the numbers of recovered single copy ortholog genes against a known ortholog gene set from a close related taxon group (using for example BUSCO [98], CEGMA [99] or the recent webserver gVolante [100]) can be difficult for venom gland tissue [90]. For specific gland tissue transcriptomes, less ortholog genes are to be expected compared to multi or body tissue transcriptomes, which reflect rather complete orthologous gene sets of a species. Furthermore, genome data of closely related organisms are often missing, especially for neglected organisms, to define meaningful orthologous gene sets. In most cases, venom gland transcriptomics is applied using de novo assembly because no complementary genome (the same species of which the transcriptome is sequenced) and no reference genome from a closely related species is available. The general picture is that the field of genomics in venomics still needs to grow and only a few genomes of venomous species are currently available [4]. To cover details on genomics is not a goal here; however, it must be clear that, without a genome backbone, the power of de novo transcriptomics remains restricted and interpretations of the results should be made with caution. In contrast to a comparative assembly approach that maps reads against genome data as backbone, a de novo assembly still remains after all NP-hard, which means that no efficient computational solution is known [101,102].

Holding et al. conclude that more reliable results are achieved by comparing and potentially combining assemblies from different assemblers that apply different k-mer sizes [90]. This approach shows one future direction for transcriptomics in evolutionary venomics. A clear piece of advice is that, instead of analyzing only one assembly, different assemblies with different software programs and settings (including kmer ranges) should be performed for a most reliable recovery of toxin transcripts. The contigs can then be merged based on similarity by available cluster tools (for example cd-hit [103]), resulting in one comprehensive assembly.

**Figure 3:**
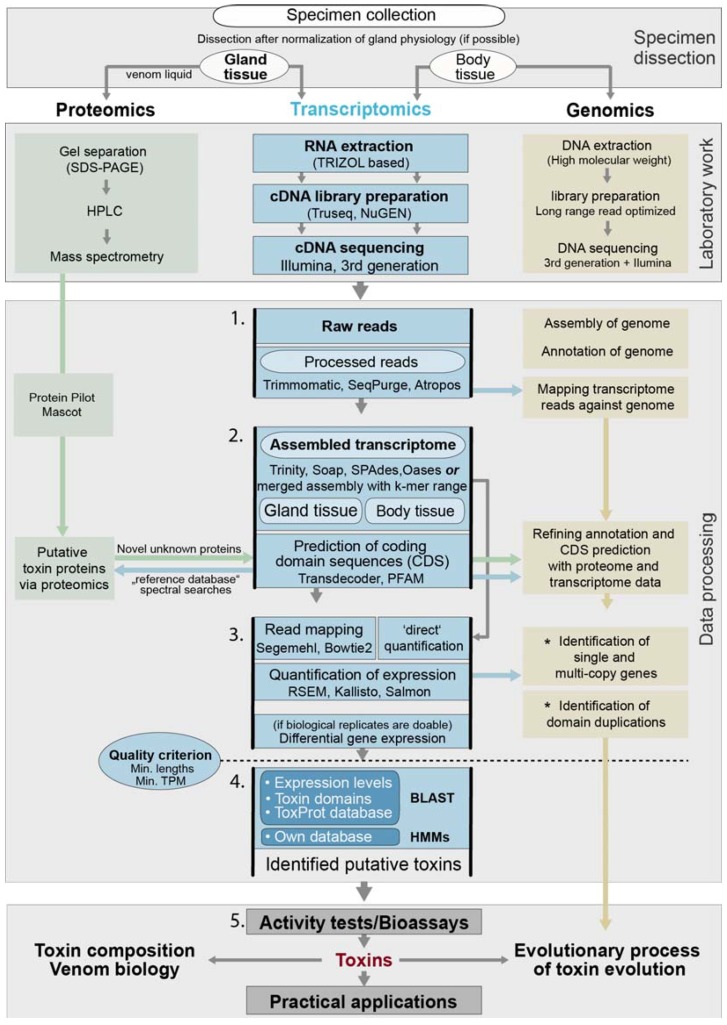
General workflow for RNASeq in modern evolutionary venomics. Different analysis steps that are discussed in more detail are numbered. Arrows highlight the steps in which complementary analyses and inclusion of proteomic or genomic data is important. Please note that proteomic and genomic processes are very generalized because the focus here is transcriptomics. The asterisks mark analysis steps that are adequately only to address if transcriptomics is complemented by genomic data. The methods to estimate expression levels differ between the classical read mapping and new quantification approaches (see also Section 4.4.). It is important is to consider that, only after activity tests (after step 5), candidate proteins are addressable as toxins. Before this step, they represent more or less likely putative toxins or venom proteins. The software shown is not intended to be exhaustive.

Chimeric transcripts [94,97] are of particular importance for venom studies that deal with highly similar sequence variants and represent one reason why complementary data from proteomics is so important. False positive transcripts (or isoforms) can be eliminated by focusing analyses only on transcriptome sequences that are also found in the proteome [5,53]. One strength of a high quality genome backbone is that, instead of a de novo assembly with all its complexity and possible errors, all reads can be mapped directly against the genome sequence, see Figure 3, and assembly-borne false positives are eliminated. As briefly mentioned in the previous paragraph, a future direction of de novo transcriptomics utilizing long read techniques, such as Oxford Nanopore or Pacbio SMRT [27,75,76], could overcome current difficulties in assembly, but also improve later estimation of gene expression [44]. This approach could eliminate a larger percentage of erroneous chimeric transcripts or those that are falsely created by repetitive, hard to assemble sequence fragments such as domain duplication regions. Software that performs the assembly of long transcripts with short reads is already established for genome hybrid sequencing, and further development is in progress [104,105,106,107].

### 4.3. Read Mapping

In venomics, the unknown venom composition of a species as well as the proportion of its toxin components and abundance of these transcripts are always of special interest. Since transcripts are assembled by breaking all reads into small k-mer fragments, the estimation of transcript expression levels demands basically a new assembly, but this time using a read mapping (or aligning) approach [108,109,110]. In the two-step procedure, all pre-processed reads are first back-mapped (read count) against transcripts or defined coding domain sequences (potential gene sets) predicted from the assembled transcripts [44,109]. The second step is the read quantification in a narrower sense (despite this term is not consistently used) to calculate the expression level of normalized reads for each transcript or gene model [42,44,109].

The read mapping of RNASeq data is hampered by the characteristics of transcriptome data, which makes the theory behind the approach not trivial, and results in some uncertainty about levels of mapped reads [111]. To cover the mathematical approaches behind the different strategies cannot be the scope here; for more details, please refer to the published studies on that topic [44,110,112,113]. Very briefly, sequencing errors demand that mismatches have to be allowed, but deletions and insertions also need to be addressed. Isoforms and multiple exon regions complete the list of methodological hurdles to perform an accurate read mapping. To increase the fuzziness of research in this complex topic, a vast amount of read mapping software is published (partly comparatively tested) from which one can choose [113,114,115]. An important angle to make a decision is of course the computational time and hardware that can be invested, but a more important consideration should be the theoretical approach of the applied mapping strategy. Assemblers handle multiple reads, which have several equally likely matches on multiple transcripts (for example reads that match on different isoforms) in different ways. To estimate the abundance of multiple reads, complex mathematical models were developed. Reads that map on several transcripts are either ignored, randomly assigned to one of the transcripts only or mapped to the transcript with the highest local coverage [112,116]. The read mapper Segemehl includes multiple reads by mapping and counting a read multiple times if it matches different transcripts. It can be argued that this reflects a biologically more realistic approach, but of course it impacts the resulting read numbers for transcripts, which will differ compared to other methods. The consequences for this potentially higher mapping precision are longer runtime and more sophisticated hardware requirements [112]. When analyzing toxin variants with highly similar sequences, the read mapping strategy might play an important role, but comparative studies that test more extensively its impact on transcriptome data from studies on venom evolution are missing so far.

### 4.4. Transcript Quantification and Gene Expression Level Estimation

The result of the read mapping is normally an output table with the read counts for the transcripts, which is used to quantify transcripts and calculate expression levels of identified gene models, coding domain sequences or exons [110], in our case mostly coding domain sequences of putative toxin variants. It is important that the “raw” counts cannot be compared to each other because they first need to be adjusted and normalized for transcript length and sequencing depth. Quantification tools use metrics to normalize and estimate an abundance of reads (see also Table 1). Different tools can be used for this quantification. Some allow the import of read count tables from different read mappers—for example, RSEM [117].

At this point, some general remarks about RNASeq are in order to understand the partially very complex models behind the estimation of gene expression levels. The assumption for RNASeq experiments is that fragments are sampled from transcript populations and thus, with sufficient sample size (the sequencing depth is here of major importance), one can postulate that highly expressed transcripts are also more frequently sampled and low expressed transcripts are less frequently covered [118]. Given that no (technical or biological) bias is apparent, the number of sampled reads is proportional to all (possible) transcripts that are expressed in the tissue. RNASeq measures relative amounts of RNA transcripts. Absolute transcript abundance is in general not testable but would demand comparative methods such as qRT-PCR (as the gold standard). RNASeq also does not measure gene expression per se (functional gene products) but instead the expression of transcripts. To assess gene expression via RNASeq, all possible different isoforms for each gene need to be summed up [111,118].

**Figure 4:**
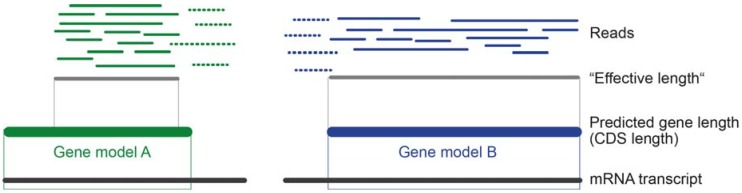
Theoretical case of read mapping for two putative toxin transcripts. Without normalization for length, gene models A and B are equally highly expressed because the same numbers of reads are mapped. Dashed reads are not counted because the applied “effective length” only includes reads that map completely within the considered coding domain sequence (CDS) coordinates. “Effective length” is simplified here based on a classic read mapping approach and thus in quotes (Mathematically, the effective length for a transcript is the mean number of start positions that are possible for reads to map with full length within that transcript (for more details see [111,118])).

The transcript length plays an important role because the probability to sample longer transcripts is by chance higher than short ones, simply because more reads map on longer transcripts [109]. To avoid misinterpretation of similar read numbers for shorter and longer transcripts, read length needs to be accounted for. If the number of counted reads is similar to a longer transcript, a shorter transcript is more highly expressed (see Figure 4).

For a proportional interpretation of transcript expression levels, the sequencing depth needs to be included to see the relation of mapped reads for a transcript to the overall number of reads that were sequenced. When working with de novo transcriptomes of neglected organisms, it can be expected that the prediction of gene models is not very precise, as most genes are predicted based on model organisms and their genome data. Depending on the applied method to predict coding domain sequences (CDS), some reads map only fragmentally onto the predicted CDS (see Figure 4). In this case, reads are by default in most cases dropped.

Commonly used statistic metrics or units to reflect transcript and gene abundance (please notice that transcript and gene are not the same) are FPKM (fragments per kilobase million) [119] and TPM (transcripts per million) [111,120] (for an overview, see Table 1). Both metrics normalize for transcript lengths and for the library size but differ in the order of doing that. TPM, however, is favorable over FPKM because first the read length is normalized and then the sequencing depth. Short but highly expressed transcripts normally receive extremely high FPKM values [120]. The normalization of read length as a first step when TPM is calculated compensates this effect. An isoform that is expressed in the same amount in two samples will show different FPKM values if other transcripts’ expression is changed and the mean expressed transcript length differs. That makes FPKM unusable and inconsistent among samples [120]. TPMs of transcripts sum up and reflect the same proportions within samples but also between samples in contrast to FPKM. However, it must be clear that both TPM and FPKM are actually not designed to compare transcript abundance between samples because they reflect relative and not absolute abundances. In some cases, a careful statement about TPMs of a specific protein that occurs in two different samples (for example body and venom gland tissue) can be justified—for example, if its relative abundance (or expression magnitude) compared to other fractions within a sample is discussed (Protein X is the most highly expressed class in both sample A and sample B).

A basic assumption to compare transcript abundance and proportion between samples is that different sample conditions (for example different tissue types) also result in different populations of transcripts, for which the proportions are not directly comparable [121]. If specific conditions force an overexpression of specific transcripts, these might additionally skew the analyses towards one experiment. This effect is enhanced if the transcripts are expressed in only one sample. In this case, genes that are in reality similarly highly expressed in both samples A and B are underestimated for sample B if sample B includes several other, unique and highly expressed transcripts. A statistical framework to compensate this condition based on the transcriptome raw data is, for example, the trimmed mean of *M*-values (TMM) method [121,122]. Very briefly explained, TMM is based on the calculation of log-fold changes of medium expressed genes that are used as scaling factors, which are then incorporated into the analyses. For a comparison between TMM and two other methods to quantify read abundance between samples (applicable without replicates), see the work by Maza [122]. Particularly when working on small, neglected organisms, the collection of sufficient tissue of venom glands is often not possible (see previous paragraphs). Thus, in most cases, replicates are not present and expression levels are normally discussed for single, but pooled samples. It has to be stated that the results of any analysis with no replicates needs to be interpreted with caution because the statistical framework and power of a differentially expressed gene analysis (with several replicates) is not given.

Alternative methods, mostly now referred to as “RNA quantification”, estimate transcript and gene expression without performing the previously described “classical” read mapping alignment and counting approach. Recent studies indicate that these alignment-free software tools like Sailfish [123], Kallisto [124] or Salmon [125] might outperform read mapping and counting not only time-wise, but in particular seem to perform better when estimating expression levels in the case of multiple isoforms. Application of both methods and a final comparison of received quantification levels to test the robustness of identified putative toxins (by similar proportions of values) might be a way to avoid analyses and discussions of toxin compositions being misled on method induced bias or errors in the expression level analysis.

In most cases, the collectable material of small, neglected organisms is not sufficient to design a multi-replicate strategy. However, when enough material of venom gland tissue from different specimens (pooled or single individuals) is present, several cDNA libraries can be preparated with a minimum of three biological replicates (in contrast to technical replicates that imply that one sample is sequenced multiple times). After the sequencing, a classical differential gene expression approach can be applied using R based script packages such as DEseq or EdgeR [42,117,121,126]. In particular, the software Corset is designed for de novo transcriptome based gene expression [127]. Depending on the applied statistical method used to estimate the expression levels, the mathematical models and assumptions are becoming very complex to include multi-mapping reads [111,118] and estimations of expected counts. Most complex algorithms further operate with an approximation of effective fragment length, which is (in very general terms) the transcript length but in relation to the effective (overall) length (or possible starting positions) of reads that map within the transcript [111,118]. However, differential gene expression is beyond the scope of this review and likely only in rare cases to apply for smaller, neglected organisms.

## 5. Identification of Putative Toxins

### 5.1. First Thresholds to Prevent False Positive Transcripts

Before putative toxins are identified, several considerations about quality control and thresholds should be carefully reflected. Toxins are injected in most cases into another organism via a venom delivery system and are thus expressed in this structure in addition to house keeping genes and non-toxin related physiologically ‘normal’ genes that constitute the transcriptome.

To minimize false positive hits, all transcripts that are being discussed as putative toxins should match sequences that were identified in complementary proteome data and represent the secretome or “crude venom”. A usual workflow is that, in a first step, transcriptomic and proteomic analyses are independently conducted. Then, in a second step, the RNASeq assembly is additionally used as a “reference database” to identify proteome sequences and to assist the identification of novel peptides from neglected organisms based on the transcriptome, see also Figure 3. In a third step, an iterative hmmer search based on identified transcript and protein sequences could be applied to identify all possible transcript variants [34]. This might be important if specific protein classes are subjects of a study. The settings for a confident identification of sequences via proteomics depend a lot on the used platform. Most frequently used software packages that perform statistical tests to guarantee the robustness of proteome results are ProteinPilot (AB SCIEX, Concord, ON, Canada) or Mascot (Matrix Science Ltd., London, UK). Both programs internally assign confidence scores based on the number of high-confidence peptide sequences. Additionally, false discovery rates can be used estimated from decoy-based searches. A strict setting is, for example, an allowed false discovery rate (FDR) of 1%. The details of proteomcis are not the focus here, so please refer for further details to relevant proteomics publications, e.g., [6,53].

Finally, only those transcripts that feature a signal peptide by a search against the SignalP database [128] should be discussed. Depending on the sequencing depth, one can expect an almost complete coverage of transcripts. This also of course includes unwanted sequences, for example from contamination (for small organisms a clean dissection of the venom delivery system might be difficult). Transcriptome sequences derived from body tissue might help to separate gland unique venom proteins that generally could represent interesting putative toxin candidates. However, the power of comparative body tissue to identify unique venom gland proteins is limited. For centipedes and robber flies, it was recently shown that many venom gland protein sequences actually also match sequences in the body tissue [5,53]. In most cases, the expression levels of proteins in the glands were significantly higher, so a strategy to filter for highly expressed transcripts is recommended (see also the next Section 5.2.). Please note that the advantage of RNAseq to cover peptides/proteins that are missed or underestimated by proteomics is briefly discussed separately in Section 6.1.

Linking to the applied sequencing depth decisions on technical thresholds, such as minimum transcript lengths or TPM values, might be reflected upon too. The expectation that toxins should be higher expressed in venom delivery systems is a general one, but the consequence is that low expressed transcripts could be erroneous transcripts or not part of the venom proteins (even if they are identified in the proteome). In relation to the expression of major venom proteins, a threshold to exclude these low expressed variants is more conservative but might prevent further inclusion of false positive putative toxin transcripts.

### 5.2. Different Strategies to Identify Putative Toxins

Many methods to estimate expression levels already include protein database searches to predict CDS regions for each transcript, for example against Pfam [129]. However, a specific identification of putative toxins is normally performed in more detail after assembly, quantification and annotation to refine the prediction of possible toxins or venom proteins.

A commonly used strategy to identify putative toxins in (unspecified) transcripts is to BLAST against known toxins from public databases, for example the UniProt ToxProt knowledgebase, in which venom protein data is integrated and toxins are manually curated [130]. Interestingly, BLAST search related bias might occur if only BLAST-P is used. In some cases, it seems that transcripts are more complete reported and better annotated using BLAST-N [131]. A consideration to prevent false positives is to restrict database sequences to identify possible toxin transcript to “gold standard” toxins only, for which the toxicity or activity is known and empirically tested. Many sequences in UniProt represent predicted venom proteins or identified putative toxins based on similarity, but often they derive from body tissue transcriptome or are DNA based genome sequences from model organisms, which can mislead toxin identification. However, novel putative toxins are hard to identify by a too strict approach, and less strict settings that allow matches to proteins labeled as similar to or predicted as venom protein or toxin, might have to be applied (see also later paragraph on strategies to handle novel proteins). A relatively new database re-using UniProt is VenomKB, a database that tries to provide a centralized resource for venoms also including a novel venom ontology [132,133]. ToxClassifier utilizes a machine learning approach to train HMMs (see next paragraph) that discriminate toxins from other proteins but for a broad spectrum of taxa [134]. A few independent databases are more taxon specific and might be of interest in cases where toxins of the particular group are targeted. Examples are Arachnoserver [135] for spider toxins and ConoServer [136] for cone-snail toxins.

Often, known toxins are searched in transcriptome data using the hmmer3 tool [137]. Based on alignments that include several sequences of a specific toxin, a Hidden Markov Model (HMM) profile is built that predicts the probability for each position of new sequences (from the transcriptome) to match the profile based on observation of each position of the present alignment. HMMs are also utilized to predict and annotate proteins in general protein (family) databases, for example Pfam [129,138]. One disadvantage of this very fast and precise method is that many sequences are necessary to train the HMMs. Often, only one or few sequences are available for specific or rather recently described toxins. In these cases, an HMM profile is meaningless because, in order to calculate reliable probabilities based on observations for a state or position for each position, as many sequences as possible are needed. However, a bypass could be the implemented jackhammer routine in hmmer3.2 that performs an iterative search and builds profiles from target sequences if they pass the chosen threshold [137].

Novel and uncharacterized venom proteins are generally difficult to identify by transcriptomics, particular for neglected organisms. In case of a quite unique organism, of which related taxa are represented only with low coverage in databases, the transcript annotation becomes challenging. One possibility to screen for novels is to filter for high read abundance and to validate if highly expressed, uncharacterized transcripts are present [5]. Motif search and matches against proteome data might enable a further characterization. An ultimate approach is of course the synthesis and subsequent activity-tests of these novel, putative toxins. In particular, novel candidates are most interesting from an applied perspective for activity and bioassay pipelines as potential, taxon unique venom proteins that could harbor new functions or activities.

## 6. Key Advantages of RNASeq

### 6.1. Small but Mighty –RNASeq Covers Smaller Peptides That are Missed by Proteomics

Transcriptome data estimates abundances of small peptides more appropriately in cases where proteomic methods fail to detect smaller molecules. This bias seems to originate in proteomically detection issues. One example is given by Rokyta and Ward for scorpion venom typical AMPS (Antimicrobial peptides) [39]. That bioactive proteins in some instances are more reliably reaped from transcriptome-only data has substantial consequences for applied research if bioactive peptides or proteins such as AMPs are mined, particularly in smaller and neglected organisms [139,140]. Transcriptome data is easy to generate and to screen for targets with, for example, highly specific hmmer searches (see Section 5.2). Target sequences can be synthesized and tested subsequently in a second step for bioassays and activity tests [139]. This approach is extendable to other venom proteins of interest.

### 6.2. With Great Power Comes Great Responsibility—Transcriptome-Only Data

One major advantage of RNASeq data is that it currently provides the most established and straightforward way to assess venom composition and relative transcript abundance of venom components. More importantly, for many very small organisms, RNASeq represents the only way to gain insights into their venoms [44,141] based on venom gland transcriptomics. The small sizes or rare abundance of these species prevent collectable venom quantities to be sufficient for a thorough proteomic analysis. RNASeq remains, in these cases, the only possibility for those putative venom proteins to be studied. However, when utilizing a transcriptome only approach, all aforementioned limitations apply.

## 7. Conclusions and Perspectives

Transcriptomics— or RNASeq—is a powerful tool to pre-screen for putative toxins in venomous species and is almost indispensable to estimate and compare relative abundances of venom components and different expression levels of toxins. It constitutes an important method to study or characterize, on a first level, interesting venoms, particularly from smaller, not easy to access neglected organisms. In combination with complementary proteomics, activity tests or bioassays, the transcriptomically screened and identified venom proteins and putative toxins can then be further characterized. However, one pitfall is that, without critical data analyses, unintentional over interpretation of data can be easily introduced. In this overview, several steps are critically discussed and suggestions made to provide a guide that helps to prevent avoidable errors. General recommendations are not easy to give because different decisions need to be made on a case by case basis. One general obvious conclusion is that, for a reliable identification of toxin transcripts, more computational power and time has to be invested from the start, in particular to preprocess and assemble the data. A final combination of RNASeq data with proteomics is to be aspired to whenever possible.

In a longer perspective, the current snapshots of expressed venom proteins via transcriptomics (and complementary proteomics) need to be extended to understand how venoms—as most important evolutionary adaptations—evolve in organisms. More neglected taxa need to be studied with the full methodological triangle in evolutionary venomics to draw a more detailed, robust picture of venom evolution. For many smaller, neglected organisms, the strategy to analyze protein expression from multiple tissue samples comparatively to venom glands is often not possible due to their sizes. Recent studies show that this broader approach gives several new insights about possible ancestral toxin variants and processes of toxin evolution [142,143]. The generation of complementary genome data (which demands also transcriptomes from multiple tissues) is not only indispensable for overcoming current limitations by de novo transcriptomics, but also to provide a better understanding of fundamental processes that drive toxin evolution [4]. Currently, just a few studies address general mechanisms and processes of toxin evolution that are only comprehensible with genome backbones [14,143,144,145,146,147]. The new insights can finally be extended to assess in depth the physiological networks that are involved in venom synthesis. Last but not least, the functional morphology of venom delivery systems need to be studied in more detail, as many toxins are, for example, potentially expressed in different structures of the venom glands [5,37,65,72]. Maybe a consortium initiative similar to those from other fields, as mentioned in the Introduction, could spearhead and coordinate the progress in such a complex field like evolutionary venomics.

## Figures and Tables

**Table 1:** Used metrics to compare and normalize read counts of transcripts or genes within samples. The meaning, formula and calculation steps for each metric are given. *T* in the formula stands for Transcript.

Metric	Meaning and Formula (Source)	More Detailed Description and Calculation Steps
Read count	Read number estimated for a transcript	This reflects the “raw” read number per transcript, which is given as first result by most read mappers
CPM	Read number counts per million	This is the read count normalized by the number of sequenced reads (library size).
RPKM	Reads per kilobase (kb) per million. Reads are normalized with library size and then read length. $\frac{Reads\;for\;T_{x}}{\left(\frac{Length\;of\;T_{x}}{10^{3}}\right)*\;\left(\frac{Total\;of\;Reads}{10^{6}}\right)}$	(1) Total reads are divided by 1,000,000 to scale per million. (2) Mapped reads are divided by the scaling factor normalizing for sequencing depth resulting in reads per million. (3) Reads per million are divided by the transcript length (in kb).
FPKM	Fragments per kilobase (kb) per million.	Same as RPKM, but paired ends are taken into account, in case a fragment occurs in both reads it is only counted once.
TPM	Transcripts per million. Transcripts are normalized with read length first and then by the number of read numbers of the library. $\left(\frac{Reads\;for\;T_{x}}{Length\;of\;T_{x}}\right)*\frac{1}{\sum \frac{Reads\;for\;T_{all}}{Length\;for\;T_{all}\;}}*10^{6}$	(1) Mapped reads are divided by transcript length (in kb) resulting in reads per kb. (2) All reads per kb values are counted up and divided by 1,000,000 to receive a per million scaling factor. (3) The reads per kb are finally divided by the scaling factor.
